# Whole-Genome Sequencing Applied to the Molecular Epidemiology of Shiga Toxin-Producing *Escherichia coli* O157:H7 in Argentina

**DOI:** 10.1128/genomeA.01341-16

**Published:** 2016-12-01

**Authors:** Claudia Carolina Carbonari, Nahuel Fittipaldi, Sarah Teatero, Taryn B. T. Athey, Luis Pianciola, Marcelo Masana, Roberto G. Melano, Marta Rivas, Isabel Chinen

**Affiliations:** a Servicio Fisiopatogenia, Departamento Bacteriología, Instituto Nacional de Enfermedades Infecciosas-ANLIS “Dr. Carlos G. Malbrán,” Buenos Aires, Argentina; b Public Health Ontario, Toronto Laboratories, Toronto, Ontario, Canada; c Department of Laboratory Medicine and Pathobiology, University of Toronto, Toronto, Ontario, Canada; d Subsecretaría de Salud, Laboratorio Central, Neuquén, Argentina; e Instituto Tecnología de Alimentos, Centro de Investigación de Agroindustria, Instituto Nacional de Tecnología Agropecuaria (INTA), Morón, Argentina

## Abstract

Shiga toxin-producing *Escherichia coli* strains are worldwide associated with sporadic human infections and outbreaks. In this work, we report the availability of high-quality draft whole-genome sequences for 19 O157:H7 strains isolated in Argentina.

## GENOME ANNOUNCEMENT

Shiga toxin-producing *Escherichia coli* (STEC) strains are a common cause of both sporadic infections and food and waterborne outbreaks. In Argentina, hemolytic uremic syndrome (HUS) is endemic with approximately 400 new cases reported annually.

STEC O157:H7 is the dominant serotype and *stx*_2a_/*stx*_2c_ the prevalent genotype. The main source of O157 is cattle. Previous studies have shown that O157:H7 strains belonging to clade 8 and lineage I/II by lineage specific polymorphism assay (LSPA-6) are prevalent in human and cattle isolates. Also, it has been demonstrated that *stx*_2a_ strains belonging to clade 8 were high toxin producers (1, 2).

This work reports the availability of high-quality draft whole-genome sequences for 19 (11 human and eight bovine) O157:H7 strains isolated in Argentina. The isolates had previously been characterized phenotypically and genotypically by PFGE, single nucleotide polymorphism (SNP), and LSPA-6 (3, 4).

Strains were grown overnight on Tryptic soy agar (BD-Difco, Le Pont de Claix, France), and genomic DNA extraction (QIAamp DNA Mini Kit, Qiagen Group) and quantification (Qubit fluorometer, Invitrogen, Eugene, Oregon, USA) were performed according to the manufacturer protocols. Libraries were prepared using the Nextera XT DNA library preparation kit (Illumina, San Diego, CA, USA), and sequenced as paired-end reads (150 bp + 150 bp) on an Illumina MiSeq instrument (Illumina 1.9, San Diego, CA, USA). Quality of raw reads was assessed with FastQC v0.11.5 (5) and custom scripts. Paired-end reads were then *de novo* assembled using the A5 pipeline (6) and obtained contigs reordered against reference strain TW14359 (3) using Mauve v 2.3.1 (7). The sequences were annotated with the NCBI Prokaryotic Genomes Automatic Annotation Pipeline (8).

Further analysis of the draft genomes will be included in a future publication.

### Accession number(s).

This whole-genome shotgun project has been deposited at DDBJ/ENA/GenBank under the BioProject number PRJNA299801. The versions described in this paper are the first draft genome sequences for the 19 STEC O157:H7 strains, under the accession numbers listed in Table 1.

**TABLE 1:** Accession numbers and assembly metrics of the annotated STEC O157:H7 strains

Isolate no.	Origin	City or province/yr of isolation	NCBI accession no.	No. of scaffolds	Genome size (bp)	*N*_50_	G+C content (%)
GN1209	*Homo sapiens*	La Pampa/2006	LXJJ00000000	112	5,322,179	201,363	50
GN1210	*Homo sapiens*	Buenos Aires/2008	LXJI00000000	117	5,431,510	267,470	50
GN1211	*Homo sapiens*	Buenos Aires/2007	LXJH00000000	117	5,303,086	159,713	51
GN1212	*Bos taurus*	Buenos Aires/2007	LXJG00000000	126	5,320,347	185,674	50
GN1213	*Homo sapiens*	Buenos Aires/2006	LXJF00000000	143	5,349,522	170,212	50
GN1214	*Bos taurus*	Buenos Aires/2007	LXJE00000000	162	5,361,881	243,406	50
GN1215	*Homo sapiens*	Buenos Aires/2007	LXJD00000000	151	5,427,792	171,358	51
GN1216	*Bos taurus*	Buenos Aires/2008	LXJC00000000	137	5,378,586	237,563	50
GN1217	*Homo sapiens*	Chubut/2006	LXJB00000000	158	5,476,251	200,755	50
GN1218	*Bos taurus*	Santa Fe/2007	LXJA00000000	170	5,473,683	179,381	50
GN1219	*Homo sapiens*	Chubut/2007	LXIZ00000000	123	5,428,694	200,826	51
GN1220	*Bos taurus*	Buenos Aires/2007	LXIY00000000	135	5,441,312	180,736	50
GN1221	*Homo sapiens*	Buenos Aires/2002	LXIX00000000	114	5,328,396	237,941	50
GN1222	*Bos taurus*	Santa Fe/2007	LXIW00000000	140	5,334,541	170,808	50
GN1223	*Homo sapiens*	Santa Fe/2008	LXIV00000000	105	5,300,977	248,557	50
GN1224	*Bos taurus*	Buenos Aires/2006	LXIU00000000	116	5,238,450	196,625	50
GN1225	*Homo sapiens*	Buenos Aires/2007	LXIT00000000	145	5,417,114	201,254	50
GN1226	*Homo sapiens*	Rio Negro/2007	LXIS00000000	116	5,298,547	171,490	50
GN1227	*Bos taurus*	Corrientes/2007	LXIR00000000	132	5,312,947	201,355	50
